# Early loss of bone mineral density is correlated with a gain of fat mass in patients starting a protease inhibitor containing regimen: the prospective Lipotrip study

**DOI:** 10.1186/1471-2334-13-293

**Published:** 2013-06-28

**Authors:** Eric Bonnet, Jean-Bernard Ruidavets, Anne Genoux, Laurence Mabile, Florian Busato, Martine Obadia, François Prévoteau, Bruno Marchou, Patrice Massip, Fabrice Marion-Latard, Cyrille Delpierre, Jacques Bernard, Bertrand Perret

**Affiliations:** 1 Hôpital Joseph Ducuing, Infectiologie, Toulouse, F 31000, France; 2 INSERM, U1048, Toulouse, F 31300, France; 3 Département Epidémiologie, CHU Toulouse, Toulouse, F 31300, France; 4 CHU Toulouse, Hôpital Purpt fédératif de Biologie, Toulouse, F 31300, France; 5 CH Tarbes, Service des Maladies Infectieuses, Tarbes, F 64000, France; 6 CHU Toulouse, Hôpital Purpan, Service des Maladies Infectieuses et Tropicales, Toulouse, F 31300, France; 7 CHU Toulouse, Hôpital La Grave, Service de Dermato-vénérologie, Toulouse, F 31000, France; 8 CHU Toulouse, Hôpital Larrey, Service de Médecine du Sport, Toulouse, F 31300, France

**Keywords:** HIV, Antiretroviral treatment (ART), Osteopenia, Osteoporosis, Bone mineral density, Bone metabolism, Lipodystrophy, Dyslipidaemia

## Abstract

**Background:**

HIV-infected patients starting antiretroviral treatment (ART) experience deep and early disorders in fat and bone metabolism, leading to concomitant changes in fat mass and bone mineral density.

**Methods:**

We conducted a prospective study in treatment-naive HIV-infected patients randomized to receive two nucleoside reverse transcriptase inhibitors in combination with either a protease inhibitor (PI) or a non-nucleosidic reverse transcriptase inhibitor (NNRTI), to evaluate early changes in body composition, bone mineral density and metabolic markers as differentially induced by antiretroviral therapies. We measured changes in markers of carbohydrate, of fat and bone metabolism, and, using dual-emission X-ray absorptiometry (DXA), body composition and bone mineral density (BMD). Complete data on changes between baseline and after 21 months treatment were available for 35 patients (16 in the PI group and 19 in the NNRTI group).

**Results:**

A significant gain in BMI and in total and lower limb fat mass was recorded only in patients receiving PI. A loss of lumbar BMD was observed in both groups, being higher with PI. Plasma markers of bone metabolism (alkaline phosphatase, osteocalcin, collagen crosslaps) and levels of parathormone and of 1,25diOH-vitamin D3 significantly increased in both groups, concomitant with a decline in 25OH-vitamin D3. Lipids and glucose levels increased in both groups but rise in triglyceride was more pronounced with PI. A correlation between loss of BMD and gain of fat mass is observed in patients starting PI.

**Conclusions:**

We evidenced an early effect of ART on lipid and bone metabolisms. PI lead to a significant gain in fat mass correlated with a sharp drop in BMD but active bone remodelling is evident with all antiretroviral treatments, associated with low vitamin D levels and hyperparathyroidism. In parallel, signs of metabolic restoration are evident. However, early increases in lean and fat mass, triglycerides, waist circumference and leptin are much more pronounced with PI.

## Background

Selective effects of nucleoside reverse transcriptase inhibitors (NRTI) and of protease inhibitors (PI) on adipose tissue differentiation and on lipid metabolism have been described [1,2]. More recently, attention was focused on loss of bone mineral density (BMD) recorded in HIV-infected patients [3]. Prevalence of osteoporosis and osteopenia was found about twice higher in HIV-patients than in seronegative controls [4,5]. In a large population-based study, occurrence of pathological fractures was more frequent in HIV-patients [6]. Several studies have also documented low levels of vitamin D in HIV-patients, while fragments of bone collagen degradation were found elevated [7-10]. However, contrasted observations have been made regarding other markers, like parathormone or osteocalcin [7,8]. A few longitudinal studies have addressed the issue of bone loss, but patients were already treated at baseline and time of follow-up was very variable, leading to contradictory conclusions [11,12]. In order to monitor early changes in body composition and BMD, we implemented a prospective study – LIPOTRIP -, following up treatment-naive HIV-infected patients, randomized to receive either a non-nucleosidic reverse transcriptase inhibitor (NNRTI) or a PI-based regimen. In parallel, we evaluated the changes in markers of lipid and bone metabolism and looked for correlations between changes in body composition and BMD. This study enables the description of differential effects of treatments on BMD, body composition and metabolic changes.

## Methods

A prospective study was set up, following up naive HIV-infected patients commencing ART, enrolled between January 2004 and April 2006. Using a random number table, they were assigned to either one of two therapeutic schemes: first group patients were treated with two NRTIs and a ritonavir-boosted PI (“PI group”) while second group patients received two NRTIs and a NNRTI (“NNRTI group”). Physicians had some recommendations for preferred regimens: ZDV-3TC + EFV in NNRTI group and ZDV-3TC + any PI in the PI group. However, at the time the study started fosamprenavir / ritonavir was extensively used.

**Figure 1:**
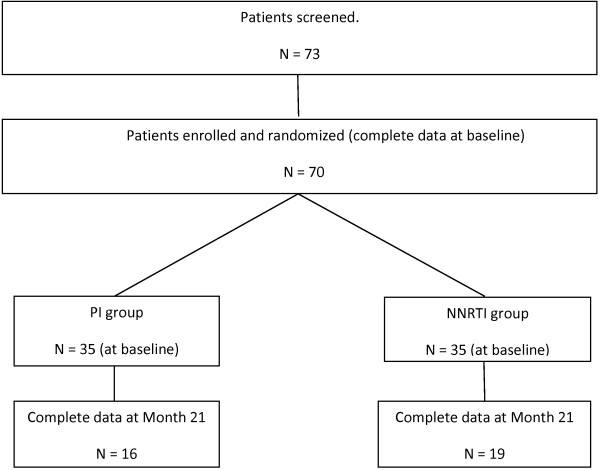
Patients screened, enrolled, randomized, and analyzed in the study.

Inclusion criteria were adult HIV-infected patients (age > 18 years) naive of ART. Patients with current or recent (< 1 month) opportunistic diseases were excluded from the study as were those with diabetes mellitus, known dyslipidaemia, alcoholism, obesity, Cushing syndrome, thyroid disease, menopause, corticosteroid treatment and anyone who could not sustain an 8-hour fasting. All patients had signed informed consent and the protocol was approved by the local Ethics Committee (“Protection Committee of people for Biomedical Research”, Toulouse 1. France). At baseline and every three months thereafter, clinical and biological data were collected, including weight and BMI, CD4-cells and other blood cell counts, HIVviral load, haemoglobin level, ionogram and creatinine concentration, biomarkers of carbohydrate, lipid and bone metabolism. Body composition was evaluated by DXA at baseline and after 9 (M9) and 21 months (M21) on treatment. Seventy three patients were screened and 70 were enrolled in the study. Complete data at baseline, 9 and 21 months were available for 35 patients, as shown in Figure 1).

The relatively small proportion of patients for whom complete data were available at 21 months can be largely explained by the constraints of laboratory tests including a glucose tolerance test and because the site where the DXA was performed was quite distant from the consultation site.

### Biological measurements

Blood was collected after an overnight fast, CD4 lymphocytes were determined by flow cytometry and plasma HIV viral load was measured by quantitative PCR. Serum glucose, triglycerides, cholesterol and high-density lipoprotein (HDL)-cholesterol were assayed with enzymatic reagents on an automated analyzer (Roche Diagnostics, Meylan, France). Low-density lipoprotein (LDL)-cholesterol was calculated using the Friedwald equation. Serum lactate was assayed in an automated analyzer (Olympus, Les Ulis, France), as were activities of total alkaline phosphatase (ALP) and γ-glutamyltranspeptidase (γGT) assayed at 37°C. Pyruvate was assayed with an enzymatic method. Albumin concentration was determined by a direct colorimetric assay (Olympus). Osteocalcin and β-crosslaps, representing the C-ter telopeptides of type I collagen, and PTH (1–84) were measured by two-site immunoassays using electro-chemiluminescence signals in a Roche analyzer. The 25- hydroxyl-vitamin D3 (25-OH D3) was determined using an automated immunoassay (Liaison, DiaSorin, Les Ulis, France), cross-reacting with the parent 25-OH-vitamin D2. Bone ALP and 1,25-dihydroxyl-vitamin D3 (1,25-diOH D3) were measured by specific radioimmunoassay (Becton-Dickinson, Marseille, France). Insulin was measured by an immunoassay using a chemi-luminescence signal on an automated analyzer (Advia-Centaur, Siemens HealthCare Systems). Leptin was measured by a specific radioimmunoassay (Millipore Corp, St. Charles, Missouri, USA).

### Densitometric measurements

All determinations were done with the same device: the DPX-L (Lunar Corporation®), and acquisition and analysis software 4.6. We determined total lean and fat masses, lower limb lean and fat masses, trunk lean and fat masses and fat mass ratio (FMR), defined as the ratio of % trunk fat mass over % leg fat mass. Values of BMD obtained from measurements at the hip and whole body were found to be congruent for the general population. Thus, in order to simplify analyses by DXA, we decided to measure whole body BMD, as an indicator of BMD in cortical bone. We also measured BMD at the lumbar spine (L2-L4) in the two groups, reflecting changes in cancellous bone.

### Statistical analysis

#### Baseline comparisons

In bivariate analyses, chi-square test was used to compare the distribution of qualitative variables between treatment groups. When asymptotic chi-square was not appropriate, the Fischer’s exact test was performed. Mean values of continuous variables were compared by Student’s t-test. The Shapiro-Wilks and Levene’s tests were used to check the normality of the distribution of residuals and the homogeneity of variances, respectively. When basic assumptions were not satisfied for Student’s t-test, a logarithmic transformation of the variable was done. Log-transformed variables were used for all subsequent analysis.

#### Multivariate analyses

Subjects were measured over time as repeated measurements data. We used linear mixed effects models to fit response trends over time and to determine treatment group effects (i.e. interaction of treatment X period). Random intercept and random slope were taken into account to model heterogeneity in intercepts and in slopes of the individual’s own regression line for repeated measurements data. The treatment x period interaction was systematically sought for each parameter analysed and was considered as statistically significant for p <0.10. A stratified analysis by treatment group was conducted to test specific changes over time. A systematic adjustment for age, sex and geographical origin of individuals was done. The level of significance was set at p < 0.05. Statistical analyses were performed using the SAS statistical software, release 9.1 (SAS Institute Inc, Cary, North Carolina, U.S.A.).

## Results

### Baseline characteristics of patients according to treatment groups

Naive HIV-infected patients were randomly assigned to either one of two therapeutic schemes. All patients started a treatment containing two NRTIs: zidovudine + lamivudine [51.5%], tenofovir + emtricitabine (or lamivudine) [20.6%], didanosine + lamivudine (or emtricitabine) [14.7%], abacavir + lamivudine [13.2%], these frequencies being similar in the two treatment groups. In the PI group, patients received the following drugs: fosamprenavir [54.3%], atazanavir [20%], saquinavir [11.4%], indinavir [8.6%], lopinavir [5.7%], all being boosted with ritonavir. In the NNRTI group they received either efavirenz [68.6%] or nevirapine [25.7%]. Complete data (including DXA results and all biological parameters at 9 and 21 months) were available from 35 subjects, who did not switch ART during the study period and were equally distributed in the two treatment groups. The number of patients lost to follow-up was similar in the 2 groups, and baseline data were identical for patients lost to follow-up and those included in the final analysis (data not shown) (Table 1).

**Table 1:** Baseline characteristics of patients according to treatment groups: clinical, immunovirological and body composition data, biological markers

**Parameter**	***+ PI (n = 16)***	***+ NNRTI (n = 19)***	***p***	***Normal range***
Age, y	42.3 (14.5)*	39.7 (11.9)*	0.57	
Sex ratio (M,%)	62.5	63.2	0.96	
Origin			0.96	
European (%)	75.0	73.7		
African (%)	25.0	26.3		
Current smokers (%)	0	31.6	0.03	
Duration of diagnosed infection, years	2.2 (2.9)*	1.6 (1.8)*	0.46	
CDC categories			0.31	
A + B (%)	81.3	94.7		
C (%)	18.7	5.3		
Viral load (log10/ml)	5.05 (0.85)*	5.04 (0.55)*	0.97	
CD4 lymphocytes/ml	240 (153)*	240 (131)*	0.90	
BMI, kg/m^2^	23.8 (3.6)*	24.0 (3.6)*	0.84	
Body Lean Mass, kg	46.3 (8.8)*	49.4 (10.3)*	0.35	
Body Fat Mass, kg	18.4 (8.9)*	19.4 (10.9)*	0.79	
Fat Mass ratio	0.97	0.97	0.99	
In men	1.05 (0.16)*	1.03 (0.14)*	0.81	≤ 1.5
In women	0.85 (0.10)*	0.87 (0.14)*	0.74	≤ 1.2
BMD L2-L4 (g/cm^2^)	1.233	1.249	0.82	
Cholesterol, g/l	1.88 (0.45)*	1.61 (0.34)*	*0.05*	1.5 – 2.2
HDL-cholesterol, g/l	0.44 (0.08)*	0.38 (0.10)*	*0.10*	> 0.4
Triglycerides, g/l	1.14 (0.58)*	0.99 (0.48)*	*0.46*	0.5 – 1.5
Glucose, g/l	0.84 (0.08)*	0.83 (0.09)*	*0.79*	0.72 - .1.05
Total alkaline phosphatase, U/l	168 (37)*	173 (49)*	*0.73*	100 – 280
Bone alkaline phosphatase, μg/l	8.1 (3.2)*	8.3 (2.6)*	*0.88*	5 – 20
β-Cross-laps, μg/l	353 (186)*	302 (161)*	*0.39*	160 – 440
25-OH-vitamine D3, μg/l	20.7 (8.2)*	19.4 (12.0)*	*0.71*	22 – 45
1,25-di-OH-vitaminne D3, ng/l	49.1 (18.7)*	49.3 (18.4)*	*0.97*	25 – 60
Parathormone, ng/l	34.1 (12.9)*	35.1 (13.3)*	*0.83*	10 - 65
Cockroft (ml/min)	83.9 (21.9)*	102.3 (17.3)*	*0.01*	

* mean value (standard deviation).

Individual characteristics are compared in Table 1. Males constituted about two thirds of the population study and mean age was 42 years. Seventy-five percent of included subjects were of European ascent and 25% of African origin. Most African patients were women (8 out of 10). Proportions of Africans and of females were similar in both groups. Duration of the diagnosed infection before ART initiation was about 2 years. Distribution among the clinical stages of the disease was similar between the two groups, with less than 20% of included patients at stage C.

Viral load at baseline was close to 5 log 10 copies/ml and average CD4 numbers was 240 in the two groups. Anthropometric measurements gave normal average values for BMI and waist circumference. Likewise, determinations of body composition indicated identical body fat mass and lean mass in the two groups. The FMR, a reliable marker of alterations of fat distribution [13], was in the normal range for both men (below 1.5) and women (less than 1.0) in the two treatment groups. Likewise, BMD in the whole body and at the lumbar spine were very close to that of the general population and similar in the two groups, as were the markers of bone differentiation (bone ALP and osteocalcin) and resorption (ß-cross-laps).

Interestingly, levels of 25-OH-D3 were similar between groups and below recommended values (> 25 μg/l). However, the active form, 1,25-dihydroxyl-vitamin D3, was in the normal range (35–70 ng/l).

Smoking habits were different, as 31% of the NNRTI group subjects were still smokers *versus* none in the PI group. Glucose, liver enzymes and alkaline phosphatase (ALP) were found normal and identical between groups, as were lactate, pyruvate, insulin and leptin (not shown). However, plasma lipids, particularly cholesterol, were somewhat lower in the NNRTI group.

### Changes in immuno-virological parameters during treatment

The two allocated therapeutic schemes were equally effective in restoring total and CD4 lymphocytes, as well as in controlling viral load (Table 2).

**Table 2:** Changes in immuno-virological, haematological and metabolic markers, anthropometric and body composition parameters, and in bone markers as a function of treatment time

***+ PI***					***+ NNRTI***				
***(n = 16)***					***(n = 19)***				
Parameter*	**Month 0**	**Month 9**	**Month 21**	***p, trend***	**Month 0**	**Month 9**	**Month 21**	***p, trend***	**p, time X treatment interaction**
CD4 lymphocytes /ml	240 (153)	376 (158)	497 (189)	*0.001*	241 (131)	445 (167)	493 (127)	*0.001*	0.54
Viral load, log10/ml	5.05 (0.85)	2.08 (0.85)	1.62 (0.07)	*0.001*	5.04 (0.55)	1.91 (0.88)	1.59 (0.01)	*0.001*	0.99
Cholesterol, g/l	1.88 (0.45)	2.22 (0.66)	2.29 (0.41)	*0.001*	1.61 (0.34)	1.95 (0.46)	2.03 (0.41)	*0.001*	*0.99*
HDL-cholesterol, g/l	0.44 (0.08)	0.45 (0.10)	0.52 (0.15)	*0.06*	0.38 (0.10)	0.52 (0.16)	0.56 (0.16)	*0.001*	***0.003***
Triglycerides, g/l	1.14 (0.58)	1.99 (1.68)	1.75 (0.82)	*0.002*	0.99 (0.48)	1.34 (1.64)	1.31 (0.97)	*0.26*	0.06
Glucose, g/l	0.84 (0.08)	0.88 (0.08)	0.87 (0.07)	*0.14*	0.83 (0.09)	0.94 (0.08)	0.90 (0.07)	*0.001*	0.08
Lactate, mmoles/l	0.93 (0.28)	1.06 (0.39)	1.11 (0.44)	*0.24*	0.98 (0.28)	0.94 (0.24)	0.93 (0.35)	*0.68*	0.19
Albumin, g/l	39.0 (4.9)	42.6 (2.0)	42.5 (2.0)	*0.006*	41.7 (3.7)	43.1 (3.5)	42.9 (2.5)	*0.07*	**0.05**
Cockroft (ml/min)	83.9	82.9	86.1	*0.73*	102.3	103.1	104	*0.66*	**0.97**
Waist circumference, cm	83.2 (11.3)	87.5 (16.7)	89.7 (13.7)	*0.04*	82.3 (7.4)	82.5 (8.3)	81.9 (9.1)	*0.51*	**0.03**
BMI, kg/m2	23.6 (3.6)	25.0 (4.9)	25.7 (5.5)	*0.02*	23.9 (3.6)	24.0 (3.7)	24.3 (3.7)	*0.73*	**0.03**
Body Mineral content, kg	3.02 (0.69)	2.97 (0.63)	2.93 (0.58)	*0.06*	3.11 (0.56)	3.08 (0.57)	3.04 (0.53)	*0.008*	0.58
L2-L4 BMD, g/cm2	1.234	1.182 (0.226)	1.186 (0.238)	*0.001*	1.249 (0.178)	1.232 (0.196)	1.230	*0.04*	**0.05**
	(0.234)						(0.176)		
Body lean mass, kg	46.3 (8.8)	48.2 (10.9)	48.9 (11.2)	*0.05*	49.4 (10.3)	49.6 (9.6)	49.8 (10.2)	*0.64*	0.09
Body fat mass, kg	18.4 (8.9)	20.2 (11.1)	21.5 (10.4)	*0.02*	19.4 (10.9)	19.5 (10.2)	19.6 (9.4)	*0.79*	**0.05**
Trunk fat mass, kg	8.87 (4.38)	9.43 (5.28)	10.28 (4.80)	*0.03*	9.03 (4.69)	9.12 (4.57)	9.49 (4.54)	*0.42*	0.19
Legs fat mass, kg	6.61 (3.39)	7.51 (4.16)	8.00 (4.24)	*0.02*	6.88 (4.18)	7.29 (4.38)	7.12 (3.70)	*0.35*	0.07
Total ALP U/l	168 (37)	193 (75)	208 (82)	*0.04*	173 (49)	227 (67)	221 (53)	*0.001*	*0.47*
Osteocalcin, μg/l	19.3 (7.6)	37.8 (15.0)	29.7 (12.8)	*0.003*	15.7 (5.1)	24.5 (8.5)	22.6 (12.6)	*0.005*	*0.19*
ß-crosslaps, μg/l	353 (186)	552 (267)	525 (240)	*0.03*	302 (161)	582 (277)	473 (226)	*0.001*	*0.56*
Calcium, mmoles/l	2.28 (0.08)	2.34 (0.10)	2.36 (0.08)	*0.02*	2.29 (0.11)	2.32 (0.15)	2.32 (0.13)	*0.31*	*0.22*
25-OH vitamine D3, μg/l	20.7 (8.2)	22.1 (9.1)	16.4 (7.2)	*0.27*	19.4 (12.0)	19.5 (12.7)	15.7 (9.6)	*0.12*	*0.81*
1,25—diOH-vit D3, ng/l	49.1 (18.7)	42.9 (8.3)	58.9 (16.6)	*0.29*	49.3 (18.4)	51.6 (17.6)	61.1 (14.7)	*0.04*	*0.54*
Parathormone, ng/l	34.1 (12.9)	36.7 (12.7)	53.4 (20.0)	*0.01*	35.1 (13.3)	47.8 (19.6)	46.5 (12.5)	*0.001*	*0.78*

* mean value (standard deviation).

### Changes in body composition and metabolic markers during treatment

**Figure 2:**
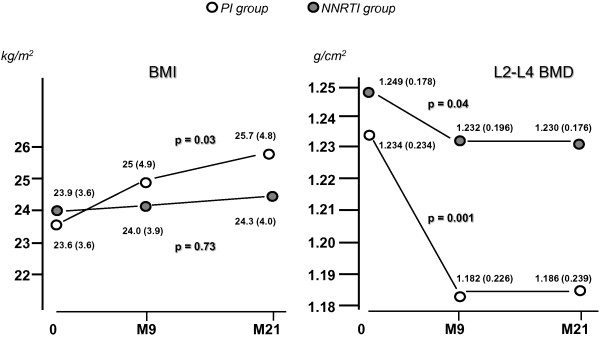
**Changes in BMI and bone mineral density as induced by two different ARV modalities.** BMI regularly increased with time, a significant 5% rise was recorded only in the PI group. Following a 21-month ART (M21) an average 3% reduction of lumbar spine BMD was observed. Bone mineral loss was different between treatments: drop in L2-L4 density was 1.5% the NNRTI group and 4% in the PI group.

**Figure 3:**
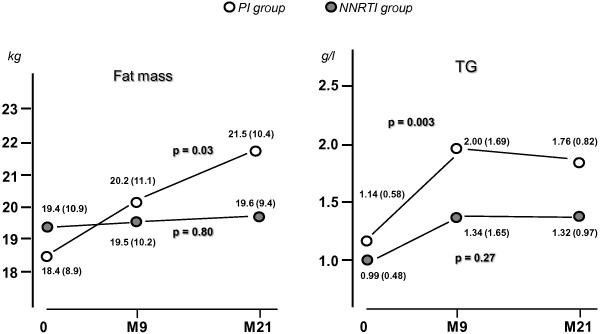
**Changes in body fat mass and in plasma triglycerides as induced by two different ARV modalities.** In the PI group, but not in the NNRTI group, there was a significant and marked rises in fat content. For triglycerides (TG), a significant rise was observed in the PI-group only, reaching levels above upper normal limits (> 1.5 g/l).

BMI regularly increased with time, significantly only in the PI group; where a 8% rise was recorded in this group (Figure 2). A parallel increase in waist circumference was also recorded in the PI group. Changes in BMI were paralleled by variations of body composition. After 21 months upon PI, lean mass was enhanced by 5% and fat mass in higher proportions (+16%). Both trunk and limbs gradually increased their fat mass with time so that the FMR was unmodified during this follow-up period (not shown). PI-sparing treatments did not lead to alterations of body composition (Figure 3). Significant rises with time were also recorded for lipids. After 21 months, total and LDL-cholesterol were enhanced by about 25%, but remained in normal ranges. HDL-cholesterol, being low at baseline, was restored to normal values with both treatments (> 0.4 g/l), yet the increase was much more pronounced in the NNRTI group. Regarding triglycerides, a significant rise was observed in the PI-group only (Figure 3), reaching levels above upper normal limits (> 1.5 g/l). Rises in plasma albumin and glucose during treatment were evidenced, being significant upon PI for albumin, and upon NNRTI for glycaemia. Finally, leptin measurements were available up to 9 months showing a differential response to treatments: leptin concentration was stable upon NNRTI (6.9 ± 7.3 μg/L at month 0 and 6.7 ± 6.8 μg/L at month 9), but sharply increased upon PI from (from 9.0 ± 10.8 to 13.8 ± 12.7 μg/L, p = 0.04).

### Changes in bone mineral density and markers of bone metabolism during treatment (Table 2)

Following a 21-month ART, an average 3% reduction of lumbar spine BMD was evident. Bone mineral loss was different between treatments: drop in L2-L4 density was 1.5% (p < 0.04) in the NNRTI group and 4% (p < 0.001) in the PI group (Figure 2). In either men or women, we found no statistical interaction between treatment time and ethnicity. Significant changes in L2-L4 BMD were also recorded when analyses were conducted separately in males (p = 0.001) and in females (p = 0.04). However, African and European women were quite different regarding basal L2-L4 BMD: 1.400 (± 0.224) versus 1.089 (± 0.169), respectively (p < 0.01). Moreover, African women experienced smaller decreases upon treatment: - 0.018 versus −0.045 g/cm2 in European counterparts (p < 0.05, not shown). It is noteworthy no woman underwent menopause during follow-up. A subanalysis combining gender and ethnicity was not possible due to the small size of subgroups. In both groups, cross-laps, representing markers of collagen degradation, were increased by 50% during follow-up. In parallel, osteocalcin, was 70% increased. Total ALP was increased in both groups and a doubling of bone ALP was recorded between baseline and month 9 (p < 0.001, not shown). Mean value for bone ALP, osteocalcin and ß-cross laps exceeded upper normal limits (see Table 1).

Serum calcium displayed a slight increase with time, significant in the PI group. Levels of 25-OH-D3 remained stable at month 9, but tented to fall by 20% during the next 12 months. Considering the whole population, this trend was close to significance (p = 0.07). Conversely, in the two groups, PTH increased significantly with time. In parallel, the active 1,25-diOH-D3 increased by 25% at 21 months, significantly in the NNRTI group. However, the difference between groups of 1,25-diOH D3 increase at 21 months was not significant (p = 0.54).

Alterations in markers of bone turnover or of calcium homeostasy were observed with both antiretroviral strategies. It is noteworthy that no patient had received vitamin D supplementation during the study period. Eight patients received tenofovir and twenty-five, did not. Changes in value of BMD, 25-OH-D3 and 1,25-diOH-D3 did not differ significantly according to whether or not patients were treated with tenofovir (data not shown).

### Correlations between changes in bone mineral density and BMI or biological markers

**Figure 4:**
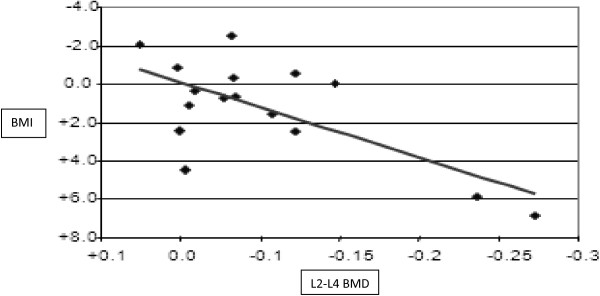
**Simultaneous variation of BMI and BMD in the PI group, between the baseline and the end of the ninth month of treatment (M9).** Changes in L2-L4 density were negatively correlated with variations of BMI (β = -0.682, 95%CI [-0.831;-0.879], p < 0.05).

In the PI group, changes in L2-L4 density were negatively correlated with variations of BMI (− 0.68, p < 0.05) (Figure 4), lean mass (r = − 0.71, p < 0.01) (data not shown) and albumin concentration (r = − 0.58, p < 0.05) (data not shown). A non-significant inverse association with trunk fat mass was also observed in the PI-group (data not shown).

This suggests that patients who experience the best metabolic restoration upon treatment are more prone to develop osteopenia. No such relationship was found in the NNRTI group. Further on, we compared simultaneous variations upon treatment of L2-L4 BMD and of various biomarkers. In both groups, changes in BMD were negatively correlated with variations of osteocalcin and β-cross-laps: bone loss was more pronounced when osteocalcin displayed the strongest increase (r = − 0.52 in the PI-group and – 0.65 in the NNRTI-group, p < 0.05) (data not shown).

## Discussion

The present study had a controlled prospective design, with treatment-naive patients randomly allocated to a first ART. It clearly shows that PI administration induces a significant decrease in lumbar spine BMD. Many cross-sectional studies and few longitudinal studies have investigated alterations of body composition in HIV-infected patients under various treatments. All those studies concluded to a low BMD in infected men and women, particularly when measured at the lumbar spine [3,12,14,15]. In a large French cohort of 35–50 years-old patients, prevalence of osteoporosis was 34% in men and 8% in women, and osteopenia was diagnosed in 50–55% [5]. Differences were also observed depending on ethnicity: osteopenia was found twice more frequent in Caucasians than in Africans [9]. Finally, a low BMD may have a clinical impact as prevalence of vertebral, hip or wrist fractures was 65% higher in HIV-patients than in healthy age-paired counterparts [6]. Components of the HIV itself, like Gp 120, might be involved in impairing osteoblast differentiation while promoting their apoptosis [16]. In the present study, baseline BMD was not lower than in the general population at the same age and in the same area. These results are not consistent with those published by others and ourselves [3,8,17-19]. These discrepancies can be explained by a shorter duration of HIV infection in the present study as compared with previous studies. It is recognised that HIV infection itself is a risk factor for osteopenia [16]. This effect seems to be correlated with the duration of infection as reported in other chronic infections (*e*.*g*. HBV and HCV infections) [20].

Regarding impact of different classes of ART, conflicting data have been reported. While some studies claimed that low BMD is observed irrespective of treatments [21], others have found a strong association with use of PI [17,22], of NRTI [12] or of Tenofovir [14]. Large meta-analyses have concluded to a 2.4-fold higher risk of osteoporosis attributable to ART [16,17]. The study by Duvivier et al. [15] including 3 groups of patients (NNRTI + PI, 2 NRTI + NNRTI, 2 NRTI + PI) also concluded to a more pronounced decrease in lumbar spine BMD in patients receiving either PI-containing regimen as compared with the NNRTI and NRTI group. Except for the presence of a third group of patients treated with PI + NNRTI, the design of this study and the characteristics of the patients and groups were fairly close to those of our study. However, the longer duration of observation in our study (90 weeks *vs* 48 weeks) enables to demonstrate that the more pronounced decrease of BMD in PI-treated patients occurred during the first months of therapy, as also shown in a prospective study comparing the effects on bone density of regimens containing atazanavir or efavirenz [23]. After the first 9 months of treatment, BMD remained stable, whether patients received PI or NNRTI. Moreover, we had measurements of markers of bone turn-over and body composition and found correlations between these variables and the BMD decrease. Bone loss following ART initiation seems to coincide with immune reconstitution, which causes a high bone resorption [24].

Although the measured variations are partly dependent on sex and ethnicity, this cannot explain the differences between our groups, as the proportions of Africans and of women were identical. However, this study confirms that BMD changes were more pronounced in Europeans than in Africans.

At a variance with changes in body composition, markers of bone build-up (bone ALP, osteocalcin) and resorption (β-cross-laps) were stimulated with either therapeutic scheme. This suggests that bone turnover is activated as soon as a minimal mineral loss has occurred. Cross-sectional studies have reported elevated levels of collagen degradation fragments and of osteoblast specific proteins in HIV-infected adults and children [25-27], particularly when treated with PI [8]. Although parameters of bone turn-over were not discriminators between treatment groups, changes in osteocalcin and cross-laps were significantly associated with the magnitude of osteopenia making them useful markers to follow-up treated HIV-patients.

Experiments in cultured cells may help to elucidate the molecular mechanisms underlying bone alterations upon ART. Regarding osteoclast differentiation, several antiretroviral molecules have demonstrated an impact on the RANK ligand (*receptor activator of NF*-*КB ligand*) pathway (RANKL) leading to its sustained activation [28,29]. Interestingly, leptin concentration sharply increased upon PI. Leptin, acting through its hypothalamic receptor, plays an indirect role in bone resorption, through stimulation of the sympathetic tone. Activation of the β2-adrenergic receptor in osteoblasts stimulates RANKL expression [30]. Moreover, increase in leptin upon PI may reflect development of peri-visceral, insulin-resistant, adipose tissue, as suggested here by the increased waist circumference. Hyperleptinemia, correlated with insulin resistance has been recently reported in HIV-patients under ART [31].

ln our study, baselevels of 25-OHD_3_ were below recommended values. Further on, at 21 months upon ART, a 20% decline in 25-OH D_3_ was recorded (p = 0.07 for the whole population). However, clinicians have not performed supplementation before the study because 25-OH D_3_ levels were not measured before the study. Supplementation was anyway an exclusion criterion. Finally, clinicians were not really aware of vitamin D deficiency at that time. Since then, a relative vitamin D deficiency (< 20 ng/ml) has been reported in a large proportion of HIV-patients, worsening after 12 months of ART [32]. In this latter study, NNRTI were incriminated in the vitamin D decline. On the other hand, several PI were shown to impair synthesis of 25-OH D_3_ and 1,25-diOH-D_3_ in cells [33,34]. A recent hypothesis suggests that ART, like other xenobiotics, may also favour vitamin D degradation, through the CYP24 hydroxylase [35,36]. Moreover, the decline of 25-OH D_3_ might up-regulate PTH synthesis [37]. In agreement with this, hyperparathyroidism has been reported upon ritonavir [38]. Apart from its role in bone resorption, PTH stimulates renal 1α-hydroxylase and has positive effects on osteoblast marker, like ALP and osteocalcin [39,40]. Thus, we may hypothesize that a low 25-OH D_3_ would trigger secondary hyperparathyroidism, which, in turn, would stimulate synthesis of 1,25-di-OH D_3_ and of osteoblast differentiation factors. Up-regulation of 1,25-di-OH D3 would be somewhat less pronounced upon PI, due to possible impact of those drugs on 25-OH D3 bioactivation [33]. Elevated calcemia would then result from increased PTH and 1,25-di-OH D3. Increased PTH might thus be a sensitive marker of bone remodelling and of vitamin D defect in treated HIV-patients.

The low metabolite concentrations observed at baseline may reflect a hypercatabolic situation in HIV-untreated patients corroborating previous observations [41]. Following ART introduction, glucose, lipids and albumin increased, likely reflecting an improvement of the energetic status associated with a better immuno-virological control. Large randomised trials have demonstrated different effects of ART on metabolic markers. A recent study showed the greatest increase in lipid parameters with a treatment combining PI and NNRTI [42]. In our study, most marked increases were for TG (+ 50%) with a specific effect of PI. PI-treated patients also experienced a homogeneous gain in fat mass both in trunk and lower limbs. Interestingly, other longitudinal studies have described increases in lean and fat masses during the first weeks upon ART, followed by a subsequent loss of limb fat after 2 years, [43,44]. Thus likely, our follow-up period was too short (< 2 years) to record any significant fat redistribution, except for a homogeneous gain. In our longitudinal study, we found that the decrease in BMD was correlated with increased BMI, the latter being mostly attributable to a gain in fat mass. We speculate that PI might switch the differentiation program of mesenchymal progenitor cells towards adipocytes, at the expanse of osteoblast formation, as already hypothesized [16].

## Conclusion

In conclusion, despite the small size of the studied population, our observations demonstrate an early effect of ART on lipid and bone metabolisms. More specifically, PI lead to a significant gain in fat mass correlated with a sharp drop in BMD but active bone remodelling is evident with all antiretroviral treatments, associated with low vitamin D levels and hyperparathyroidism. In parallel, signs of metabolic restoration are evident. However, early increases in lean and fat mass, triglycerides, waist circumference and leptin are much more pronounced with PI.

## Competing interests

The authors declare that they have no competing interests.

## Authors’ contribution

ALG and JG carried out and interpreted all biochemical assays. LM monitored the study. FML and JB conducted and interpreted all DXA. JBR and CD participated in the design of the study and performed the statistical analysis. FB, FP and MO recruited and followed almost all patients in the study. BP and EB conceived the study, and participated in its design and coordination, and drafted the manuscript. PM and BM participated in the implementation of the study, monitoring its progress and the discussion of results. All authors read and approved the final manuscript.

## Author’s information

**Author to whom reprint requests should be addressed**: Eric BONNET. Hôpital Joseph Ducuing. 15, rue Varsovie. 31000 Toulouse. France.

## Pre-publication history

The pre-publication history for this paper can be accessed here:

http://www.biomedcentral.com/1471-2334/13/293/prepub
